# Basal and Activated Calcium Sensitization Mediated by RhoA/Rho Kinase Pathway in Rats with Genetic and Salt Hypertension

**DOI:** 10.1155/2017/8029728

**Published:** 2017-01-19

**Authors:** Michal Behuliak, Michal Bencze, Ivana Vaněčková, Jaroslav Kuneš, Josef Zicha

**Affiliations:** Institute of Physiology, Czech Academy of Sciences, Prague, Czech Republic

## Abstract

Calcium sensitization mediated by RhoA/Rho kinase pathway can be evaluated either in the absence (basal calcium sensitization) or in the presence of endogenous vasoconstrictor systems (activated calcium sensitization). Our aim was to compare basal and activated calcium sensitization in three forms of experimental hypertension with increased sympathetic tone and enhanced calcium entry—spontaneously hypertensive rats (SHR), heterozygous Ren-2 transgenic rats (TGR), and salt hypertensive Dahl rats. Activated calcium sensitization was determined as blood pressure reduction induced by acute administration of Rho kinase inhibitor fasudil in conscious rats with intact sympathetic nervous system (SNS) and renin-angiotensin system (RAS). Basal calcium sensitization was studied as fasudil-dependent difference in blood pressure response to calcium channel opener BAY K8644 in rats subjected to RAS and SNS blockade. Calcium sensitization was also estimated from reduced development of isolated artery contraction by Rho kinase inhibitor Y-27632. Activated calcium sensitization was enhanced in all three hypertensive models (due to the hyperactivity of vasoconstrictor systems). In contrast, basal calcium sensitization was reduced in SHR and TGR relative to their controls, whereas it was augmented in salt-sensitive Dahl rats relative to their salt-resistant controls. Similar differences in calcium sensitization were seen in femoral arteries of SHR and Dahl rats.

## 1. Introduction

Increased vascular tone and elevated peripheral resistance are the hallmarks of human and experimental hypertension. The degree of resistance vessel constriction is determined by cytosolic calcium level and the sensitivity of contractile apparatus to it. The latter mechanism, which is called calcium sensitization, is enhancing vascular contraction at a given level of cytosolic calcium. It is partially signaled via RhoA/Rho kinase pathway that inhibits the dephosphorylation of myosin light chain through the inactivation of myosin light chain phosphatase [1–5]. Numerous studies demonstrated that the acute blockade of calcium entry through L type voltage-dependent calcium channels (L-VDCC) [6–9] or an acute attenuation of calcium sensitization by the inhibition of Rho kinase [10–13] effectively lowered blood pressure (BP), the effects being usually more pronounced in hypertensive than in normotensive rats. Nevertheless, RhoA/Rho kinase pathway is a constitutively active mechanism which is also involved in the regulation of vascular tone and BP in normotensive animals under physiological conditions [12–15].

The clinical importance of RhoA/Rho kinase pathway has been repeatedly considered in cardiovascular medicine. Its role in coronary vasospasm or pulmonary hypertension was clearly demonstrated (for review see [16]). On the other hand, the contribution of RhoA/Rho kinase signaling to the pathogenesis of human essential hypertension is less documented, although the enhanced involvement of Rho kinase in increased peripheral vascular resistance [17] and cutaneous vasoconstriction [18] was reported in hypertensive patients. Nevertheless, it should be mentioned that in rat models of type 1 or type 2 diabetes the chronic treatment with Rho kinase inhibitors ameliorated diabetic nephropathy without substantial blood pressure changes [19, 20]. Similar protective effect of chronic Rho kinase inhibition, which was not accompanied by long-term blood pressure reduction, was also demonstrated by attenuation of the damage in the heart and kidney of rats with various forms of experimental hypertension (for review see [21]).

In most models of experimental hypertension the activity of sympathetic nervous system (SNS) and/or renin-angiotensin system (RAS) is enhanced, whereas nitric oxide (NO) formation and/or availability is attenuated [22–26]. It is well known that RhoA/Rho kinase pathway-dependent calcium sensitization in vascular smooth muscle can be enhanced by numerous vasoconstrictors or attenuated by various vasodilators [2, 27]. There are two different approaches to the study of calcium sensitization which can be used in conscious animals or isolated blood vessels. The classical approach is aimed at evaluating the contribution of activated calcium sensitization to BP maintenance on the basis of BP reduction elicited by the acute Rho kinase inhibition in rats with intact endogenous vasoconstrictor systems. The in vitro analogy of this approach is the relaxation of precontracted isolated arteries by various Rho kinase inhibitors. The alternative approach is focused on basal calcium sensitization which is present in relaxed blood vessels or vascular beds devoid of major vasoconstrictor activity. Under such conditions BP increase or arterial contraction elicited by dose-dependent agonist administration is measured prior to and after Rho kinase inhibition and basal calcium sensitization is estimated from the difference between the two BP responses [28].

Using conscious rats subjected to a combined blockade of RAS and SNS, which are characterized by a very low BP, we compared BP responses to L-VDCC opening elicited by acute BAY K8644 administration at two defined levels of calcium sensitization, that is, prior to and after Rho kinase blockade by fasudil [13]. The difference between these two BP responses reflects the basal calcium sensitization since BAY K8644 treatment does not modify Rho kinase-dependent calcium sensitization [29]. This approach demonstrated a considerable attenuation of basal calcium sensitization in spontaneously hypertensive rats (SHR) characterized by enhanced calcium entry as compared to Wistar-Kyoto rats (WKY). This strain difference in calcium sensitization was confirmed by a more pronounced fasudil-induced rightward shift of norepinephrine (NE) dose-response BP curve in conscious WKY than in SHR. Furthermore, fasudil pretreatment of isolated arteries attenuated NE-induced contraction more in WKY than in SHR vessels [13]. Activated calcium sensitization was attenuated in 3-week-old prehypertensive SHR compared to age-matched WKY but was enhanced in adult SHR with established hypertension [30]. In the course of ontogenesis (studied in rats aged 3–42 weeks) the ratio between fasudil-induced and nifedipine-induced BP changes was always substantially lower in SHR than in age-matched WKY. Fasudil effects on the contractility of isolated arteries were attenuated in young but not in adult SHR [30].

The aim of the present study was to evaluate basal calcium sensitization and activated calcium sensitization in two different forms of experimental hypertension, that is, in transgenic Ren-2 (TGR) rats with genetic hypertension due to the presence of mouse Ren-2 gene and in Dahl rats with salt hypertension, because the information on calcium sensitization is lacking in both rat strains. These two hypertensive models are characterized by sympathetic hyperactivity and increased calcium entry, that is, by the enhanced BP response to acute L-VDCC blockade by nifedipine [25, 26]. Besides the abovementioned experiments performed in conscious rats, we have also examined basal calcium sensitization using femoral arteries isolated from these animal models. These results were compared with the data obtained in SHR that were studied as a reference model.

## 2. Methods

### 2.1. Animals

The animals from our breeding facility were used for all experiments. We studied male WKY and SHR aged 16 weeks as well as male normotensive Hannover Sprague Dawley (HanSD) and heterozygous Ren-2 transgenic rats (TGR) aged 18 weeks. All rats were housed under standard laboratory conditions: temperature 23 ± 1°C, 12 h light-dark cycle, ST-1 diet (containing 1% NaCl), and tap water ad libitum. In addition, we also investigated male inbred salt-sensitive (SS/Jr) and salt-resistant (SR/Jr) Dahl rats aged 20 weeks. These rats were fed a low-salt diet (0.3% NaCl) since weaning until the age of 12 weeks, when one-half of SS/Jr and SR/Jr rats was switched to a high-salt diet (5% NaCl) for 8 weeks, whereas the remaining animals were kept on a low-salt diet. All procedures and experimental protocols, which were approved by the Ethical Committee of the Institute of Physiology, Czech Academy of Sciences, conform to European Convention on Animal Protection and Guidelines on Research Animal Use.

### 2.2. Surgery

**Figure 1:**
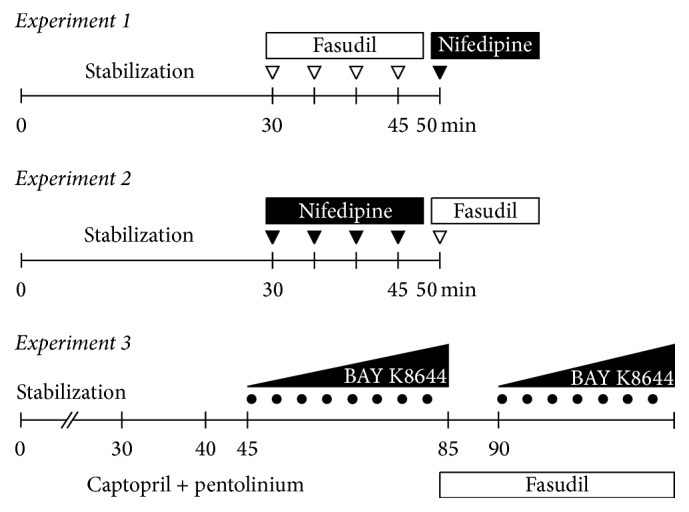
The scheme describing pharmacological interventions used in Experiments 1–3. Activated calcium sensitization was estimated from dose response to fasudil followed by the administration of a single dose of nifedipine in Experiment 1, whereas voltage-dependent calcium entry was estimated from dose response to nifedipine which was followed by the administration of a single dose of fasudil in Experiment 2. Basal calcium sensitization was measured as a fasudil-dependent difference in BP elevation induced by dose-dependent administration of calcium channel opener BAY K8644. The blockade of RAS and SNS preceded dose response to BAY K8644 measured prior to and after fasudil infusion in Experiment 3.

For BP measurement and drug application, polyethylene catheters were inserted into the left carotid artery (PE50) and jugular vein (PE10) one day before the experiment [13, 25, 30]. The experiments were carried out in conscious rats kept in small transparent cages. BP was measured using PowerLab system (ADInstruments, Bella Vista, NSW, Australia) between 8 : 00 AM and 11:30 AM to reduce circadian variations in BP levels. All animals were allowed to stabilize for a period of 30 min before any experimental protocol was performed. Separate age-matched groups of rats were used for each experimental protocol described below. The scheme of Experiments 1–3 is depicted in Figure 1.

### 2.3. Experiment 1: Activated Calcium Sensitization in BP Maintenance

The level of activated calcium sensitization was estimated from the extent of BP reduction induced by acute fasudil administration to conscious rats with intact RAS and SNS. Following a 30 min stabilization period, cumulative doses of Rho kinase inhibitor fasudil (1, 2, 4, and again 4 mg/kg) were administered intravenously to determine the role of RhoA/Rho kinase pathway in BP maintenance. Thereafter a single dose of nifedipine (0.75 mg/kg) was administered to fasudil-pretreated rats to evaluate calcium entry at low level of calcium sensitization [13, 30].

### 2.4. Experiment 2: Voltage-Dependent Calcium Entry in BP Maintenance

The level of voltage-dependent calcium entry was estimated from the extent of BP reduction induced by acute nifedipine administration to conscious rats with intact RAS and SNS. Following a 30 min stabilization period, cumulative doses of L-VDCC blocker nifedipine (0.05, 0.1, 0.2, and 0.4 mg/kg) were administered intravenously to determine the role of these calcium channels in BP maintenance. Thereafter a single dose of fasudil (10 mg/kg) was administered to nifedipine-pretreated rats to evaluate calcium sensitization at low level of calcium entry [23, 25, 30].

### 2.5. Experiment 3: Basal Calcium Sensitization in Conscious Rats

Basal calcium sensitization was measured as a fasudil-dependent difference in BP elevation induced by dose-dependent administration of calcium channel opener BAY K8644. After the stabilization period, rats were subjected to the intravenous RAS blockade by captopril (10 mg/kg) and SNS blockade by pentolinium (5 mg/kg) to eliminate the endogenous angiotensin II-dependent and sympathetic vasoconstriction [23]. Thereafter L-VDCC agonist BAY K8644 was administered intravenously in increasing noncumulative doses (0.1–80 *μ*g/kg) in the absence and in the presence of Rho kinase inhibitor fasudil (10 mg/kg bolus followed by 10 mg/kg/h infusion) [13]. BP changes were calculated as BP increases above BP level established after the combined RAS plus SNS blockade and additional Rho kinase inhibition, respectively. BP response to BAY K8644 represents vasoconstriction elicited by the enhancement of calcium entry at the basal level of calcium sensitization, whereas BP response to BAY K8644 after fasudil pretreatment corresponds to vasoconstriction induced by increasing calcium entry which occurs at calcium sensitization considerably attenuated by Rho kinase inhibition. Thus the degree of basal calcium sensitization can be estimated from the difference in BAY K8644-induced BP responses recorded prior to and after fasudil treatment. On the other hand, BP response to BAY K8644 in the presence of fasudil reflects calcium entry through actually opened L-VDCC [28].

### 2.6. Experiment 4: Basal Calcium Sensitization in Isolated Arteries

Basal calcium sensitization was estimated from the reduction in the development of isolated artery contraction by Rho kinase inhibition. Endothelium-denuded femoral arteries were isolated from all studied groups of rats. Their segments (~2 mm long) were placed in a Mulvany-Halpern isometric myograph (620 M, DMT, Denmark) and incubated in modified Krebs-Henseleit solution (KHS, mmol/l: 119 NaCl, 4.7 KCl, 1.17 MgSO_4_, 25 NaHCO_3_, 1.18 KH_2_PO_4_, 2.5 CaCl_2_, and 2 g/l glucose; 37°C; bubbled with 95% O_2_ and 5% CO_2_). A cumulative concentration-response curve to phenylephrine (10^−8^ to 3.10^−4^ mol/l) was determined in the absence and in the presence of Rho kinase inhibitor Y-27632 (10 *μ*mol/l). Furthermore, a concentration-dependent K^+^-induced contraction (5–120 mmol/l) was studied before and after Rho kinase inhibition by fasudil (10 *μ*mol/l). The attenuation of arterial contractile response by both inhibitors was expressed in percentage of maximal phenylephrine- or K^+^-induced contraction.

### 2.7. Drugs

All chemicals were obtained from Sigma (St. Louis, MO, USA) except for fasudil (HA-1077, LC Laboratories, Woburn, MA, USA). Most of the drugs were dissolved in saline, whereas nifedipine and (±)-BAY K8644 were dissolved in DMSO (maximum concentration of solvent for intravenous application was 10% which per se caused no BP changes). All drugs were usually given as an intravenous bolus (1 mL/kg b.w.).

### 2.8. Statistics

Two-way repeated measures ANOVA was used to analyze BP response to cumulative doses of fasudil (Experiment 1) or nifedipine (Experiment 2) with the grouping factor of strain and repeated measures factor of drug dose. Sigmoidal four-parameter logistic model was used for fitting BAY K8644 dose-response curves through the measured values (Experiment 3). The statistical analysis was performed on the parameters of fitted curves (minimal response, maximal response, ED50, and slope) by two-way ANOVA with the grouping factor of strain and fasudil treatment (for SHR and TGR rats) or with the grouping factor of salt intake and fasudil treatment (for Dahl rats). Statistical analyses of initial MAP and MAP changes induced by acute RAS and SNS blockade (Experiment 3) and of maximal BP response (Experiments 1 and 2) and contractile response of isolated arteries to fasudil or nifedipine (Experiment 4) were performed either by one-way ANOVA followed by Fisher LSD post hoc test for multiple group comparisons (Dahl rats) or by Student's *t*-test for comparisons of two groups (SHR and TGR rats). Normal distribution of data was confirmed by Shapiro-Wilk test before further statistical analyses. The differences were considered significant at *p* < 0.05 level.

## 3. Results

### 3.1. Experiment 1: Activated Calcium Sensitization in BP Maintenance

**Figure 2:**
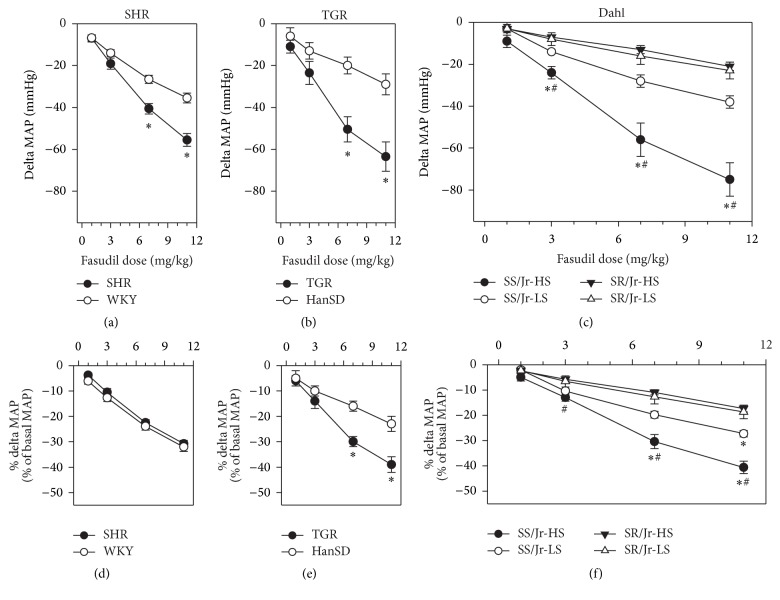
Dose-dependent MAP changes elicited by increasing cumulative doses of fasudil in rats with intact RAS and SNS: WKY and SHR rats ((a) and (d)), HanSD and TGR animals ((b) and (e)), and salt-sensitive (SS/Jr) and salt-resistant (SR/Jr) Dahl rats fed either low-salt (LS) or high-salt (HS) diet ((c) and (f)). Relative MAP changes (d, e, and f) are expressed as a percentage of initial MAP values (Experiment 1). Data are mean ± SEM; for number of rats see Table 1. ^*∗*^*p* < 0.05 versus normotensive controls (WKY, HanSD, or SR/Jr-HS); ^#^*p* < 0.05 versus corresponding rat strain fed a low-salt diet. For the statistical analysis see also Table 1.

Table 1 shows that initial MAP values were considerably increased in all three different forms of hypertension (SHR, TGR, and Dahl rats). In rats with intact endogenous RAS and SNS, the administration of fasudil caused dose-dependent blood pressure reduction in both SHR and WKY rats (Figure 2(a)) and similar findings were also obtained in TGR and HanSD rats (Figure 2(b)). In both models of genetic hypertension the absolute values of fasudil-induced MAP reduction were greater in hypertensive than in normotensive animals. When fasudil-induced MAP changes were expressed in percentage of initial MAP values, this strain difference was abolished in SHR (Figure 2(d)) but not in TGR (Figure 2(e)). There was a greater fasudil-induced MAP reduction (both absolute and relative MAP changes) in Dahl salt-sensitive than in salt-resistant rats (Figures 2(c) and 2(f)). This difference was twofold greater in salt-sensitive rats fed a high-salt diet than in those fed a low-salt diet (Figure 2(c)).

The acute Rho kinase inhibition by cumulative fasudil administration (11 mg/kg) induced substantially greater MAP reduction (delta MAP fasudil) in all three hypertensive models compared to their normotensive controls (Table 1), diminishing BP differences between normotensive and hypertensive rats (MAP after fasudil). The addition of L-VDCC blocker nifedipine following the cumulative fasudil administration (delta MAP additive nifedipine) revealed that under the conditions of decreased calcium sensitization the nifedipine-sensitive BP response was still enhanced in SHR and TGR compared to their controls but this was not the case in Dahl rats (Table 1). It is also evident that the combined Rho kinase inhibition and L-VDCC blockade further reduced BP differences between normotensive and hypertensive rats, although the resulting MAP remained to be elevated by 36% in SHR over WKY controls.

### 3.2. Experiment 2: Voltage-Dependent Calcium Entry in BP Maintenance

In all three forms of experimental hypertension the acute cumulative administration of nifedipine (0.75 mg/kg) yielded a significantly greater BP reduction compared to their corresponding controls (Table 2). Nevertheless, in normotensive WKY and HanSD rats, nifedipine elicited significantly smaller BP decrease than did fasudil and the additive fasudil lowered BP more than the additive nifedipine. A comparison of the two experiments also suggests that BP of TGR responded generally better to fasudil than to nifedipine but this was not the case of SHR (Tables 1 and 2).

When BP responses to particular inhibitors in Dahl rats studied in Experiments 1 and 2 are compared, it is evident that fasudil or nifedipine (applied as the first inhibitor) elicited similar BP changes, abolishing BP differences between hypertensive and normotensive Dahl rats. BP responses to fasudil and nifedipine applied as the additional inhibitors were also comparable (Tables 1 and 2).

### 3.3. Experiment 3: Basal Calcium Sensitization in Conscious Rats

Table 3 shows initial MAP values as well as MAP changes induced by the acute blockade of endogenous RAS (captopril) and SNS (pentolinium) in rats with three different forms of hypertension (SHR, TGR, and Dahl rats) that were used for determining BAY K8644-induced MAP changes. RAS contribution to hypertension maintenance was substantially smaller as compared to augmented SNS contribution in all three forms of experimental hypertension (Table 3).

**Figure 3:**
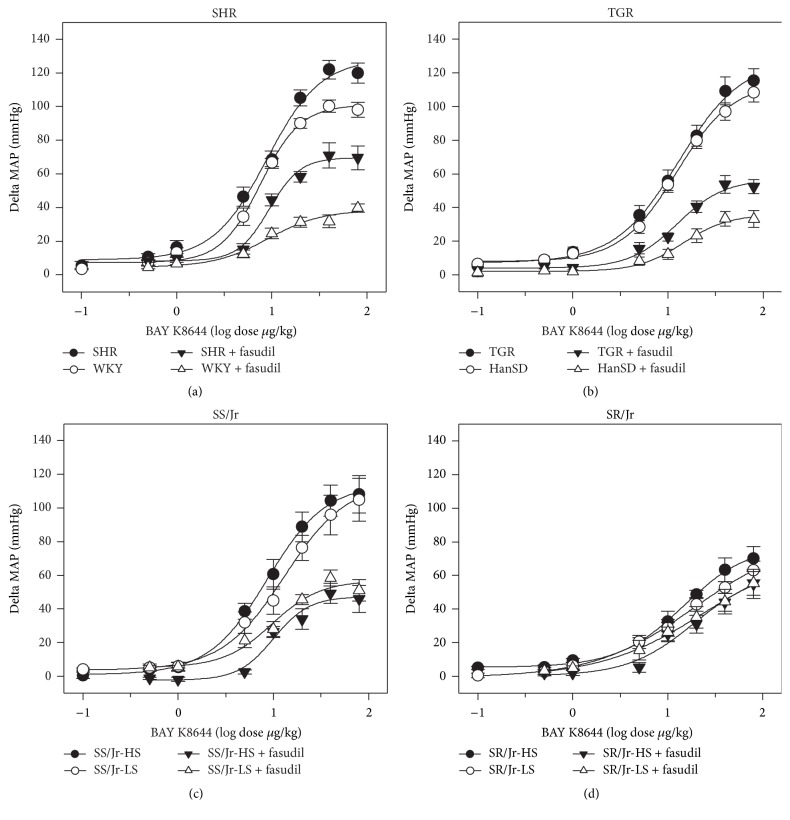
Dose-dependent MAP changes (mmHg) induced by increasing BAY K8644 doses in the absence and in the presence of fasudil in rats with inhibited RAS and SNS. (a) WKY and SHR rats, (b) HanSD and TGR animals, (c) salt-sensitive (SS/Jr) Dahl rats fed either low-salt (LS) or high-salt (HS) diet, and (d) salt-resistant (SR/Jr) animals (Experiment 3). Data are mean ± SEM; for number of rats see Table 3. The statistical analysis revealed significant differences for factors strain and treatment in maximal responses (max) of SHR, TGR, and salt-sensitive Dahl rats compared to their controls but not in other parameters (log ED50 and slope); for detailed statistical analysis see Table 4.

**Figure 4:**
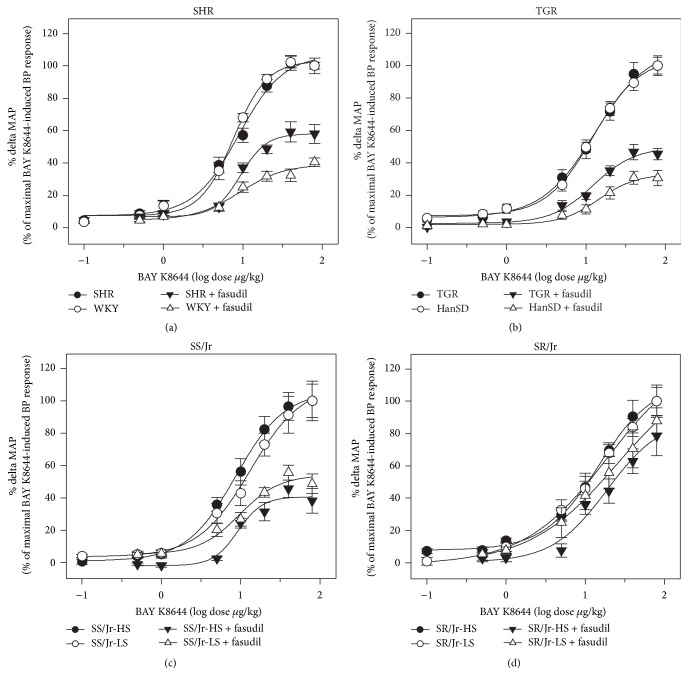
Relative BAY K8644-induced MAP changes (in percentage of maximal MAP responses) seen in the absence and in the presence of fasudil in rats with inhibited RAS and SNS. (a) WKY and SHR rats, (b) HanSD and TGR animals, (c) salt-sensitive (SS/Jr) Dahl rats fed either low-salt (LS) or high-salt (HS) diet, and (d) salt-resistant (SR/Jr) animals (Experiment 3). Data are mean ± SEM; for number of rats see Table 3. The statistical analysis revealed significant differences in relative fasudil-sensitive component of maximal MAP responses (SHR −41.9 ± 2.5 versus WKY −60.7 ± 4.2%, *p* < 0.05; TGR −50.4 ± 4.2 versus HanSD −66.9 ± 2.3%, *p* < 0.05), whereas the difference in Dahl salt-sensitive rats did not achieve statistical significance (SS/Jr-HS −59.3 ± 3.8 versus SS/Jr-LS −45.8 ± 5.9%).

The experiments were performed in the rats deprived of endogenous RAS and SNS, which were subjected to dose-dependent administration of L-VDCC agonist BAY K8644 in the absence and in the presence of Rho kinase inhibitor fasudil (Figure 3; for statistical analysis see Table 4). The presence of fasudil did not modify ED50 of BAY K8644 in any studied group of animals. It is evident that the role of fasudil-sensitive basal calcium sensitization was attenuated in SHR. In contrast, under the conditions of calcium sensitization minimized by fasudil pretreatment, the role of calcium entry through L-VDCC was more important in SHR with established hypertension than in normotensive WKY rats (Figure 3(a)). Similar findings were obtained in TGR animals which also displayed reduced contribution of basal calcium sensitization and moderately increased contribution of calcium entry to BP control as compared to HanSD controls (Figure 3(b), Table 4). The attenuation of calcium sensitization in SHR or TGR was more evident when the data were expressed in percentage of maximal BAY K8644-induced BP response (Figure 4). Nevertheless, quite different results were obtained in Dahl rats. Fasudil-sensitive calcium sensitization was substantially higher in salt-sensitive Dahl rats (Figure 3(c)) than in salt-resistant animals in which calcium sensitization was abnormally low (Figure 3(d)). High-salt intake did not cause any significant changes of basal calcium sensitization in salt-sensitive or salt-resistant Dahl rats (Figures 3 and 4).

To evaluate how much fasudil-sensitive basal calcium sensitization augments BP effects of calcium entry, we expressed BAY K8644-induced BP response as a percentage of BP response recorded in the presence of fasudil. There was a fundamental difference between spontaneous (SHR 170 ± 5% versus WKY 281 ± 13%; TGR 209 ± 6% versus HanSD 320 ± 15%; *p* < 0.001 both) and salt-induced forms of hypertension (SS/Jr-HS 234 ± 9%, SS/Jr-LS 156 ± 19 %, SR/Jr-HS 139 ± 6%, SR/Jr-LS 115 ± 3%, and *p* < 0.01). This confirms that basal RhoA/Rho kinase-dependent calcium sensitization (recorded in the absence of endogenous pressor systems) was attenuated in spontaneous hypertension but it was enhanced in salt hypertension.

### 3.4. Experiment 4: Basal Calcium Sensitization in Isolated Arteries

**Figure 5:**
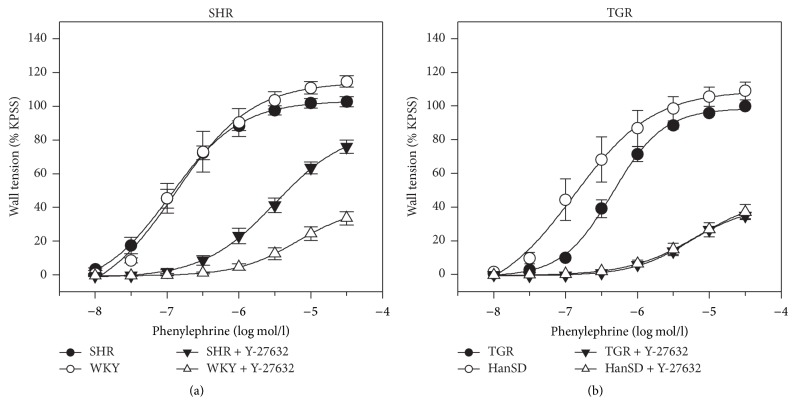
Concentration-dependent phenylephrine-induced contraction of endothelium-denuded femoral arteries isolated from WKY and SHR (a) or from HanSD and TGR (b) which were studied in the absence or presence of Rho kinase inhibitor Y-27632. Data are mean ± SEM; for number of vessels see Table 5.

Table 5 shows that both phenylephrine- and K^+^-induced contraction of endothelium-denuded femoral arteries was attenuated in the presence of Rho kinase inhibitors (Y-27632 or fasudil) in all experimental groups under study. Rho kinase inhibition caused considerably smaller effects in the arteries isolated from SHR than in those from normotensive WKY controls (Figure 5(a), Table 5). There was a tendency to the attenuated contribution of Rho kinase to arterial contractility in TGR but the difference from their HanSD controls was evident only at low phenylephrine concentrations (Figure 5(b)). On the contrary, Rho kinase inhibition elicited a greater attenuation of contractile responses to either stimulus in arteries of salt hypertensive Dahl rats (SS/Jr-HS) than in any other group of Dahl rats (Table 5).

## 4. Discussion

Our study demonstrated that basal calcium sensitization (mediated by RhoA/Rho kinase pathway), which was measured in the absence of endogenous RAS and SNS, was attenuated not only in SHR but also in TGR, which were both characterized by enhanced calcium entry through L-VDCC. In contrast, basal calcium sensitization was much greater in salt-sensitive than in salt-resistant Dahl rats, although chronic high-salt intake did not affect basal calcium sensitization in either Dahl strain. Our experiments on endothelium-denuded femoral arteries (stimulated by phenylephrine or KCl) supported our in vivo findings on the attenuation of basal calcium sensitization in SHR as well as on its augmentation in salt hypertensive Dahl rats, indicating the presence of the described calcium sensitization alterations just in vascular smooth muscle.

Furthermore, we have also employed the acute blockade of Rho kinase and/or L-VDCC in intact rats with preserved activities of RAS and SNS to evaluate the contribution of activated calcium sensitization and calcium entry to BP maintenance. It should be noted that activated calcium sensitization (measured by absolute fasudil-induced BP changes) was increased in all three models of experimental hypertension. Enhanced sympathetic nerve activity, which is characteristic for all three studied forms of experimental hypertension [26, 31, 32], is known to increase calcium entry through L-VDCC [33] as well as calcium sensitization via RhoA/Rho kinase pathway [34]. Indeed, the presence of endogenous pressor systems (SNS and RAS) augmented both calcium sensitization and calcium entry in intact rats of all strains, but this effect was more pronounced in hypertensive animals. This was especially important in salt hypertensive SS/Jr Dahl rats in which sympathetic enhancement of calcium entry and/or calcium sensitization elevated blood pressure above the level seen in SS/Jr rats fed a low-salt diet. The influence of endogenous pressor systems might be also important in essential hypertensive patients in whom the enhanced involvement of Rho kinase was reported [17, 18].

Another possible explanation for the enhanced BP response of intact hypertensive rats to fasudil or nifedipine is based upon the different baroreflex sensitivity which is usually substantially reduced in hypertensive rats compared to their normotensive controls [35–38]. Our study in intact normotensive Wistar rats [39] revealed that hypotensive action of fasudil evokes the reflex stimulation of sympathetic nerve activity which can considerably mask the full extent of fasudil-induced BP reduction by increasing their heart rate and blood pressure. Indeed, the acute ganglionic blockade by pentolinium substantially enhanced fasudil-induced BP reduction in Wistar rats pretreated with NO synthase inhibitor L-NAME [39]. Recently, we have observed that ganglionic blockade considerably augmented BP response to fasudil or nifedipine only in normotensive HanSD rats but not in hypertensive Ren-2 TGR animals [40].

There are several mechanisms which are considered to mediate BP reduction after acute Rho kinase inhibition. One of them is the activation of myosin light chain phosphatase in vascular smooth muscle leading to the dephosphorylation of myosin light chain (MLC20) [27], whereas the other mechanism is a rapid phosphorylation of endothelial NO synthase (eNOS) promoting increased NO formation [41]. The importance of the enhanced NO-dependent vasorelaxation in fasudil-induced BP reduction can be questioned by the findings that acute Rho kinase inhibition caused strong vasodilatation and BP decrease in both eNOS^−/−^ mice and rats subjected to acute or chronic blockade of NO synthase by L-NAME [12, 14]. Our own experiments [28] indicated that the acute NO synthase inhibition even potentiated BP-lowering effects of acute fasudil administration. Moreover, Rho kinase inhibition diminished the potassium- or phenylephrine-induced contraction also in endothelium-denuded arteries. Thus, BP effects of acute Rho kinase inhibition induced in vascular smooth muscle seem to be superior to those elicited in the endothelium.

BP response to acute L-VDCC blockade (e.g., by nifedipine) or acute Rho kinase inhibition (e.g., by fasudil) is usually considered to reflect solely the importance of calcium entry or calcium sensitization. However, the magnitude of BP reduction elicited by either pathway inhibitor is always dependent on the concomitant activity of the complementary pathway. Thus, the increased BP response to fasudil might be not only due to the attenuation of greater calcium sensitization at normal calcium entry but also due to the attenuation of normal calcium sensitization at greater calcium entry because the resulting BP reduction is a product of the changes in both pathways. Our study revealed that either the acute inhibition of Rho kinase by fasudil or the acute blockade of L-VDCC by nifedipine caused greater BP reduction in hypertensive rats than in their normotensive controls. The additivity of both pathways in hypertensive rats is supported by the fact that a certain fraction of their BP (15–25%) can be lowered only if the combination of nifedipine and fasudil is used. The administration of fasudil to hypertensive animals caused a greater BP decrease than the application of this drug after a previous partial BP reduction by nifedipine. The same is true for BP effects of nifedipine applied to intact or fasudil-pretreated rats (Tables 1 and 2). Our present results are in line with a strong dependence of both nifedipine-induced BP reduction [9, 32] or fasudil-induced BP decrease [28] on basal BP level.

Some in vitro studies [42, 43] suggested that RhoA/Rho kinase-dependent calcium sensitization is enhanced by simultaneous membrane depolarization and calcium release from sarcoplasmic reticulum. Sakurada et al. [42] observed that calcium-dependent activation of RhoA in contracting vascular smooth muscle can be induced either by KCl-induced membrane depolarization or by norepinephrine stimulation of G-protein-coupled receptors. Both stimuli increase active GTP-bound form of RhoA leading to vascular contraction which can be inhibited by Rho kinase inhibitors (fasudil or Y-27632). Similar inhibitory effects on KCl-induced contraction can be achieved by the absence of extracellular calcium or by L-VDCC blockade, whereas extracellular calcium removal combined with intracellular calcium store depletion causes the same inhibitory effects in norepinephrine-induced contraction [42]. Recently, Fernández-Tenorio et al. [43] reported that simultaneous L-VDCC activation and metabotropic calcium release from sarcoplasmic reticulum are required to enhance RhoA/Rho kinase activity and to elicit sustained arterial contraction. Thus, calcium channel-induced calcium release from intracellular stores plays a major role in tonic vascular smooth muscle contraction because it links membrane depolarization and L-VDCC activation with the increase in RhoA/Rho kinase-dependent calcium sensitization. It seems that chronic L-VDCC blockade can attenuate tonic vascular contraction both directly (by reducing calcium entry) and indirectly (by preventing the above calcium-induced RhoA/Rho kinase activation). These mechanisms also explain how higher sympathetic nervous activity might augment the contribution of RhoA/Rho kinase pathway to BP maintenance. This is in line with different calcium sensitization revealed in our hypertensive animals when they were studied in the presence or absence of endogenous SNS (Experiment 1 versus Experiment 3). Future experiments should evaluate the changes of calcium sensitization in animals subjected to chronic blockade of calcium entry and vice versa. It should be pointed out that the inhibition of Rho kinase activity has been reported in hypertensive patients subjected to chronic L-VDCC blockade by amlodipine [44].

It is rather difficult to describe the exact role of RhoA/Rho kinase-dependent calcium sensitization in hypertension. There is no doubt that this signaling pathway participates in the maintenance of increased peripheral resistance and high blood pressure of hypertensive animals and humans but this evidence is usually based upon the acute but not the chronic administration of Rho kinase inhibitors. In fact, chronic inhibition of Rho kinase by fasudil or Y-27632 attenuated renal and cardiac damage in rats with genetic or salt hypertension but had no long-term BP-lowering effects. This was true for SHR subjected to subtotal nephrectomy [45], DOCA-salt treatment [46], high-salt intake [47], or chronic NO synthase inhibition [48] and for salt-sensitive Dahl rats [49–52]. The absence of major antihypertensive effects of chronic Rho kinase inhibition in spontaneous or salt hypertension contrasts with the pronounced long-term BP reduction induced by chronic administration of L-VDCC blockers [53–55]. Our recent studies on the role of calcium entry and calcium sensitization in the control of vascular tone and blood pressure indicated a close cooperation of both signaling pathways in normotensive or hypertensive animals (for review see [28]). It is an open question whether a combined chronic administration of low doses of L-VDCC and Rho kinase inhibitors would have desirable antihypertensive and tissue protective effects as was suggested by the acute experiments of Porras-González et al. [56]. Long-term effects of such treatments should be examined not only in chronic experiments but also in large-scale human studies.

In conclusion, the studies with acute Rho kinase inhibition suggested the enhanced participation of RhoA/Rho kinase pathway in the maintenance of increased systemic resistance and elevated blood pressure in both experimental and human hypertension. On the other hand, chronic studies do not support such an important role of RhoA/Rho kinase in the development of experimental hypertension, although this pathway seems to be critical for the severity of end-organ damage in hypertensive animals. Our present study indicated that in the absence of sympathetic nerve activity the participation of basal Rho kinase activity in BP control is substantially different in rats with genetic or salt hypertension. In contrast, the enhanced BP reduction elicited by acute fasudil administration to intact rats with either type of experimental hypertension might be ascribed either to the augmentation of RhoA/Rho kinase signaling in hypertensive animals by their sympathetic hyperactivity or to insufficient compensation of fasudil-induced BP fall due to the impaired baroreflex efficiency in hypertensive rats.

## 5. Limitations of the Study

Our recent study [30] demonstrated several alterations of particular components of RhoA/Rho kinase pathway which are present in either prehypertensive SHR or in adult SHR animals with established hypertension. These abnormalities concern the expression of particular Rho GEFs (guanine nucleotide exchange factors) as well as the expression and phosphorylation of CPI-17 (protein kinase C potentiated inhibitor of myosin phosphatase of 17 kDa). It would be highly desirable to perform similar studies also in TGR to see the possible similarities or differences compared to SHR. However, the detailed study of the above parameters might reveal the molecular mechanisms responsible for altered RhoA/Rho kinase-dependent calcium sensitization seen in Dahl rats.

## Figures and Tables

**Table 1:** Initial values of mean arterial pressure (MAP, mm Hg) and MAP changes induced by fasudil and additional nifedipine as well as MAP levels reached after inhibitors administration in spontaneously hypertensive rats (SHR) and Wistar-Kyoto controls (WKY), heterozygous Ren-2 transgenic rats (TGR), and Hannover Sprague Dawley controls (HanSD) as well as in salt-sensitive (SS/Jr) and salt-resistant (SR/Jr) Dahl rats fed either a low-salt (LS) or high-salt (HS) diet (Experiment 1).

Strain	Number of rats	Initial MAP	Delta MAP fasudil	MAP after fasudil	Delta MAP additive nifedipine	MAP after fasudil + nifedipine
*SHR*	10	180 ± 3^*∗*^	−56 ± 3^*∗*^	124 ± 2^*∗*^	−45 ± 2^*∗*^	79 ± 2^*∗*^
*WKY*	8	110 ± 3	−35 ± 3	75 ± 2	−17 ± 2	58 ± 2

*TGR*	12	164 ± 4^*∗*^	−64 ± 7^*∗*^	101 ± 4	−35 ± 3^*∗*^	66 ± 3
*HanSD*	11	124 ± 3	−29 ± 5	95 ± 3	−25 ± 2	69 ± 2

*SS/Jr-HS *	6	182 ± 9^*∗*#^	−75 ± 8^*∗*#^	108 ± 2^*∗*^	−38 ± 3	69 ± 4
*SS/Jr-LS*	6	140 ± 7^*∗*^	−38 ± 3^*∗*^	101 ± 4	−37 ± 5	64 ± 3
*SR/Jr-HS *	8	118 ± 4	−21 ± 2	97 ± 2	−31 ± 2	66 ± 2
*SR/Jr-LS*	8	122 ± 3	−23 ± 4	99 ± 2	−32 ± 2	68 ± 1

Data are means ± SEM. Significantly different (*p* < 0.05) ^*∗*^from the respective normotensive controls and ^#^from the corresponding rat strain fed a low-salt diet.

**Table 2:** Initial values of mean arterial pressure (MAP, mmHg) and MAP changes induced by nifedipine and additional fasudil as well as MAP levels reached after inhibitors administration in WKY and SHR rats and HanSD and TGR animals as well as in SS/Jr or SR/Jr Dahl rats fed either LS or HS diet (Experiment 2).

Strain	Number of rats	Initial MAP	Delta MAP nifedipine	MAP after nifedipine	Delta MAP additive fasudil	MAP after nifedipine + fasudil
*SHR*	12	176 ± 2^*∗*^	−64 ± 3^*∗*^	112 ± 3^*∗*^	−43 ± 3	69 ± 2^*∗*^
*WKY*	9	110 ± 2	−23 ± 1	87 ± 2	−36 ± 2	52 ± 1

*TGR*	11	162 ± 5^*∗*^	−40 ± 8^*∗*^	122 ± 9^*∗*^	−48 ± 5^*∗*^	75 ± 6
*HanSD*	12	116 ± 3	−17 ± 2	102 ± 2	−36 ± 2	66 ± 2

*SS/Jr-HS *	10	188 ± 5^*∗*#^	−89 ± 6^*∗*#^	99 ± 4	−30 ± 5	69 ± 5
*SS/Jr-LS*	6	142 ± 3^*∗*^	−38 ± 4^*∗*^	104 ± 3	−37 ± 4	68 ± 3
*SR/Jr-HS *	8	120 ± 3	−24 ± 2	96 ± 3	−36 ± 2	61 ± 1
*SR/Jr-LS*	8	121 ± 5	−22 ± 3	99 ± 2	−38 ± 2	62 ± 2

Data are means ± SEM. Significantly different (*p* < 0.05) ^*∗*^from the respective normotensive controls and ^#^from the corresponding rat strain fed a low-salt diet.

**Table 3:** Initial values of mean arterial pressure (MAP, mmHg) and MAP changes induced by acute RAS blockade (captopril 10 mg/kg) and additional SNS blockade (pentolinium 5 mg/kg) as well as MAP levels after captopril and pentolinium administration in spontaneously hypertensive rats (SHR) and Wistar-Kyoto controls (WKY), heterozygous Ren-2 transgenic rats (TGR), and Hannover Sprague Dawley controls (HanSD) as well as in salt-sensitive (SS/Jr) and salt-resistant (SR/Jr) Dahl rats fed either a low-salt (LS) or high-salt (HS) diet (Experiment 3).

Strain	Number of rats	Initial MAP	Delta MAP captopril	Delta MAP pentolinium	Delta MAP captopril + pentolinium	MAP after captopril + pentolinium
*SHR*	7	176 ± 4^*∗*^	−9 ± 3	−88 ± 5^*∗*^	−98 ± 6^*∗*^	78 ± 6^*∗*^
*WKY*	6	105 ± 3	−6 ± 2	−43 ± 6	−49 ± 5	46 ± 1

*TGR*	7	175 ± 4^*∗*^	−16 ± 1^*∗*^	−81 ± 5^*∗*^	−95 ± 5^*∗*^	83 ± 4^*∗*^
*HanSD*	7	120 ± 5	−7 ± 3	−45 ± 3	−53 ± 5	70 ± 3

*SS/Jr-HS *	7	186 ± 7^*∗*#^	−18 ± 3^*∗*#^	−85 ± 8^*∗*#^	−103 ± 8^*∗*#^	84 ± 5^*∗*#^
*SS/Jr-LS*	7	140 ± 5^*∗*^	−4 ± 2	−66 ± 5	−70 ± 5	70 ± 4^*∗*^
*SR/Jr-HS *	6	126 ± 7	−4 ± 2	−69 ± 7	−73 ± 8	53 ± 2
*SR/Jr-LS*	7	117 ± 3	−5 ± 1	−58 ± 4	−63 ± 4	54 ± 4

Data are means ± SEM. Significantly different (*p* < 0.05) ^*∗*^from the respective normotensive controls and ^#^from the corresponding rat strain fed a low-salt diet.

**(a):** 

Sigmoidal logistic model parameters	Dose-response curve to BAY K8644 *before* fasudil administration		Dose-response curve to BAY K8644 *after* fasudil administration		Two-way ANOVA *p* value	
					Factor strain	Factor fasudil treatment

	*SHR*	*WKY*	*SHR + fasudil*	*WKY + fasudil*		

max	128.2 ± 7.1^*∗*^	100.9 ± 3.0	69.7 ± 2.9^*∗*#^	38.5 ± 4.1^#^	<0.001	<0.001
log ED50	0.95 ± 0.06	0.88 ± 0.03	0.97 ± 0.04	0.96 ± 0.11	ns	ns
slope	1.54 ± 0.32	2.07 ± 0.31	2.68 ± 0.65	1.54 ± 0.67	ns	ns

	*TGR*	*HanSD*	*TGR + fasudil*	*HanSD + fasudil*		

max	128.0 ± 6.5	114.0 ± 3.1	56.6 ± 4.0^*∗*#^	35.9 ± 2.5^#^	<0.001	<0.001
log ED50	1.12 ± 0.05	1.09 ± 0.03	1.10 ± 0.06	1.16 ± 0.06	ns	ns
slope	1.30 ± 0.17	1.48 ± 0.12	1.75 ± 0.40	1.83 ± 0.39	ns	ns

**(b):** 

Sigmoidal logistic model parameters	Dose-response curve to BAY K8644 *before* fasudil administration		Dose-response curve to BAY K8644 *after* fasudil administration		Two-way ANOVA *p* value	
					Factor salt intake	Factor fasudil treatment

	*SS/Jr-HS*	*SS/Jr-LS*	*SS/Jr-HS + fasudil*	*SS/Jr-LS + fasudil*		

max	114.3 ± 3.2	115.4 ± 7.2	47.4 ± 4.3^#^	56.9 ± 6.2^#^	ns	<0.001
log ED50	0.94 ± 0.03	1.12 ± 0.06	1.00 ± 0.08	0.99 ± 0.11	ns	ns
slope	1.41 ± 0.13	1.32 ± 0.20	2.32 ± 0.87	1.71 ± 0.76	ns	ns

	*SR/Jr-HS*	*SR/Jr-LS*	*SR/Jr-HS + fasudil*	*SR/Jr-LS + fasudil*		

max	78.4 ± 3.3	81.9 ± 5.7	62.9 ± 15.2	77.0 ± 12.6	ns	ns
log ED50	1.17 ± 0.04	1.27 ± 0.08	1.28 ± 0.20	1.40 ± 0.17	ns	ns
slope	1.26 ± 0.12	0.83 ± 0.08	1.25 ± 0.57	0.79 ± 0.17	ns	ns

Data are means ± SEM. max: maximal response (mmHg), log ED50: median effective dose (log dose *µ*g/kg), and slope: slope of the dose-response curve (mmHg/ log dose *µ*g/kg). Significantly different (*p* < 0.05) ^*∗*^from the respective normotensive controls and ^#^from the corresponding rats measured before fasudil administration.

**Table 5:** Maximal contractile responses of endothelium-denuded femoral arteries stimulated by either phenylephrine (Phe, 3.10^−4^ mol/l) or potassium (K^+^, 120 mmol/l) and the attenuation of these contractile responses by Rho kinase inhibitors Y-27632 or fasudil in blood vessels isolated from spontaneously hypertensive rats (SHR) and Wistar-Kyoto controls (WKY), from heterozygous Ren-2 transgenic rats (TGR) and Hannover Sprague Dawley controls (HanSD) as well as from salt-sensitive (SS/Jr) and salt-resistant (SR/Jr) Dahl rats fed either a low-salt (LS) or high-salt (HS) diet (Experiment 4).

Strain	Number of segments	Maximal Phe-induced contraction (mN/mm)	% change induced by Y-27632	Number of segments	Maximal K^+^-induced contraction (mN/mm)	% change induced by fasudil
*SHR*	10	9.4 ± 0.2	−26 ± 5^*∗*^	11	10.8 ± 0.4^*∗*^	−34 ± 3^*∗*^
*WKY*	6	8.8 ± 0.5	−67 ± 3	7	9.4 ± 0.4	−45 ± 3

*TGR*	6	10.8 ± 0.4	−65 ± 2	12	12.9 ± 0.4^*∗*^	−50 ± 3
*HanSD*	6	12.0 ± 0.7	−67 ± 3	12	10.9 ± 0.6	−43 ± 4

*SS/Jr-HS *	11	9.9 ± 0.5^#^	−64 ± 2^*∗*#^	8	13.5 ± 0.7	−60 ± 6^*∗*#^
*SS/Jr-LS*	10	12.3 ± 0.7	−34 ± 3^*∗*^	8	14.0 ± 0.8	−34 ± 4
*SR/Jr-HS *	8	10.0 ± 0.5	−50 ± 2	9	14.3 ± 1.0	−33 ± 3
*SR/Jr-LS*	8	11.4 ± 0.9	−50 ± 4	8	13.3 ± 1.0	−35 ± 2

Data are means ± SEM. Significantly different (*p* < 0.05) ^*∗*^from the respective normotensive controls and ^#^from the corresponding rat strain fed a low-salt diet.
